# Cell-free plasma hemoglobin removal by dialyzers with various permeability profiles

**DOI:** 10.1038/srep16367

**Published:** 2015-11-10

**Authors:** Michael Hulko, Melanie Kunz, Mehmet Yildirim, Sandra Homeyer, Oliver Amon, Bernd Krause

**Affiliations:** 1 Gambro Dialysatoren GmbH (part of Baxter International Inc.), Research & Development, 72379 Hechingen, Germany; 2 University Hospital Tuebingen, Pediatric Nephrology, 72076 Tuebingen, Germany

## Abstract

The release of hemoglobin from mechanically stressed erythrocytes into plasma is a general side effect of extracorporeal therapies, such as extracorporeal membrane oxygenation or hemodialysis. In many reported cases dialysis patients showed elevated cell-free plasma hemoglobin (CPH) levels which are associated with pathophysiological effects. In this *in vitro* study, the CPH clearance capacity of various filters with different permeability profiles was measured. Simulated dialysis treatments were conducted and clearance was calculated from variations in CPH concentrations over time by measuring plasma absorbance at 405 nm. Conventional high-flux filters exhibited no detectable clearance of CPH. High-flux filters with extended permeability exhibited clearances between 5.8 ± 1.2 and 12.7 ± 1.7 ml/min when tested with plasma and between 5.8 ± 1.2 and 11.3 ± 1.6 ml/min when tested with whole blood. septeX high-cutoff filters had clearances between 13.8 ± 1.8 and 15.5 ± 1.7 ml/min when tested with plasma and of 22.6 ± 2.9 ml/min when tested with whole blood. This study demonstrated that filters with extended permeability and the septeX filter enable CPH removal when used as in chronic and acute settings.

The release of hemoglobin from mechanically stressed erythrocytes into plasma is a general side effect of extracorporeal therapies^1^,^2^. Methods to remove cell-free plasma hemoglobin (CPH) from plasma are rarely described even though insights into the pathophysiological effect of hemoglobin are growing^3^,^4^,^5^,^6^,^7^.

The erythrocyte membrane can become damaged and hemoglobin is then released from the erythrocyte compartment into the plasma. The causes of erythrocyte damage include hereditary disease^8^ and chemically or mechanically induced hemolysis^1^,^2^. Mechanically induced hemolysis occurs when shear forces act on the erythrocytes such that the membrane ruptures. During extracorporeal blood purification, high shear forces can occur when flow characteristics change rapidly at, e.g., the vascular access point, a peristaltic blood pump, sites of stagnant flow, or kinked blood lines. Extracorporeal blood flow cannot be avoided in extracorporeal blood purification therapies; consequently, CPH levels are elevated by such treatments.

Hemoglobin is a tetrameric protein with a molecular weight of 62.6 kD and is composed of 2 α and 2 β subunits (Uniprot^9^ accession number P69905 and P68871). The tetramer is in equilibrium with the αβ dimer^10^,^11^ while further dissociation into monomers is hardly detectable under physiologic conditions^12^,^13^. In the body, hemoglobin is tightly confined to the intracellular compartments of erythrocytes. CPH concentrations for healthy individuals is in the range between 6 and 34 mg/L^14^. Hemoglobin is removed from plasma by binding to the hemoglobin scavenger protein haptoglobin, followed by the recognition of this complex by CD163 on the surface of monocytes, internalization by endocytosis and finally degradation^15^. The binding capacity of haptoglobin for hemoglobin is 0.7–1.5 g/L^5^.

Acute episodes of mechanical hemolysis have been reported as a side effect in pediatric patients during extracorporeal membrane oxygenation (ECMO) with CPH concentrations up to 2.05 g/L^16^. At such concentrations, the capacity of the haptoglobin scavenging system is exceeded, and adverse outcomes were associated with elevated levels of CPH. Elevated CPH concentrations have been reported for continuous venovenous renal replacement circuits under certain circumstances^17^.

In chronic hemodialysis (HD), acute episodes of mechanical hemolysis are rarely reported and were mainly caused by inappropriate application of therapy equipment^1^. However, CPH can be chronically elevated at concentrations substantially above the normal range^18^,^19^. In a study with 14 HD patients the baseline CPH concentration was 196 ± 43 mg/L and increased to 285 ± 109 mg/L during HD treatment^19^. This increase was related to acutely blunted endothelial function, which was measured using flow-mediated dilation after a single HD session.

In this *in vitro* study, the CPH clearance capacity of various hemodialysis filters with different permeability profiles was analyzed to explore possibilities of CPH removal.

## Methods

### Dialyzers

We evaluated seven types of dialyzers with membranes of different pore size and representing different permeability profiles: (P170H) Polyflux 170H 1.7 m^2^ with a high-flux membrane (Gambro Dialysatoren GmbH, Hechingen, Germany), (CorDiax) FX CorDiax80 1.8 m^2^ with a high-flux membrane (Fresenius Medical Care, Bad Homburg, Germany), (MCO1-4) four different types of prototype dialyzers 1.8 m^2^ with a high-flux membrane with extended permeability (Gambro Dialysatoren GmbH, Hechingen, Germany), and septeX 1.1 m^2^ with a high-cutoff membrane (Gambro Dialysatoren GmbH, Hechingen, Germany). The MCO1-4 prototype dialyzers are based on the Polyflux technology with a membrane permeability between conventional high-flux and high-cutoff membranes as defined by dextran sieving characteristics^20^. Within the different types of MCO prototypes the permeability increased from MCO1 to MCO4^21^. Further membrane characteristics of the dialyzers used in the study are summarized in Table 1 and are taken from the respective data sheets or were measured according standard ISO 8637:2010.

### Simulated dialysis treatments with blood or plasma

Dialysis treatments were simulated on commercial monitor systems with a closed loop recirculation circuit on the blood side; the dialysis fluid was prepared by the monitor system from standard concentrates. Blood-side flow rate (QB), dialysate-side flow rate (QD), and ultrafiltration rate (UF) were controlled by the respective monitor systems. The system was primed with saline solution prior to the recirculation of either 1 L of heparin-anticoagulated bovine whole blood from a local slaughterhouse (Balingen, Germany) or of 1 L of bovine plasma (Kraeber & Co., Ellerbek, Germany) whereby no animal was sacrificed for the sole purpose of blood collection. The total protein content in each test was 60 ± 5 g/L, and in tests with blood, the hematocrit was 32 ± 3%. The test solution was maintained thermostatically at 37 °C throughout the experiment in a closed container. CPH was generated by adding 6 ml of freeze-thawed bovine whole blood to the test medium. Samples were collected at the start of the experiment and after 5, 20, 40 and 60 min. In one experiment, CPH was generated by adding human blood instead of bovine blood. Heparinized human whole blood was collected under medical supervision from healthy donors. The experiments and procedures were in accordance with ethical guidelines approved by Gambro Dialysatoren GmbH (part of Baxter International Inc.).

septeX sets were tested on the Prismaflex monitor system (Gambro Lundia AB, Lund, Sweden) with bovine blood or plasma and under 2 different flow conditions: QB 200 ml/min, QD 42 ml/min (2.5 L/h), UF 0 ml/min and QB 200 ml/min, QD 133 ml/min (8 L/h), UF 0 ml/min. There was a plasma control run at QB 200 ml/min, QD 42 ml/min, UF 0 ml/min at which addition of hemoglobin was omitted.

P170H, CorDiax, and MCO1-4 were tested on the AK 200 Ultra S monitor system (Gambro Lundia AB, Lund, Sweden) with bovine blood and QB 400 ml/min, QD 500 ml/min, UF 0 ml/min and with bovine plasma and QB 400 ml/min, QD 700 ml/min, UF 0 ml/min. Tests of P170H, MCO1-4 and CorDiax with plasma had an initial recirculation phase of 60 min with closed dialysate ports prior to start of the experiment and hemoglobin was added after 55 min of initial recirculation. There was a plasma control run with MCO4 where addition of hemoglobin was omitted. During experiments with the AK 200 Ultra S monitor system continuous fluid removal from the test solution was consistently observed. The amount of removed fluid was 8% of the total fluid volume on average. Consequently, clearance values are underestimated by about 8%. On the Prismaflex monitor system the volume balance error was below 2%.

### Hemoglobin determination

Hemoglobin was determined in 2 ways: Absorbance was measured photometrically at 405 nm (A_405_) using an Ultra Microplate Reader EL808 (BioTek Instruments GmbH, Bad Friedrichshall, Germany) detecting the Soret adsorption band of the heme molecule of hemoglobin. For better comparability of A_405_ time course plots the values were normalized such that A_405_ at the beginning of the experiment was set to 1. Mass concentrations were measured as a cyanide complex using the Hemoglobin FS Reagent Kit (Diasys Diagnostic Systems GmbH, Holzheim, Germany) and an Ultrospec 6300 (GE Healthcare, Frankfurt, Germany) spectrophotometer at 540 nm and a kit-specific conversion factor according to manufacturer’s instructions. Measuring A_405_ was used for clearance calculations because of the sensitivity of the method. The Hemoglobin FS Reagent Kit was used to verify the specificity of the A_405_ measurements and to determine absolute hemoglobin concentrations. In each case the plasma samples were prepared by centrifuging blood samples and collecting the supernatant.

### Clearance calculation

Clearance was calculated based on first-order kinetics for the variation in the plasma concentration in a single compartment model^22^ as a function of time according to


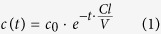


where *c*(*t*) is the concentration at time *t, c*_0_ is the initial concentration, *t* is the time in min, *Cl* is the clearance in ml/min, and *V* is the total plasma volume in ml.

For clearance calculation equation (1) was transformed to


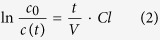


with A_405_ at the start of the experiment for *c*_0_. t was the time of the sampling points after 5, 20, 40 and 60 min and A_405_ at the sampling points was taken for *c*(*t*) accordingly. ln [*c*_0_/*c*(*t*)] was plotted against t/V and the clearance was calculated as the slope of a linear regression.

### Calculation of removed hemoglobin mass and reduction ratios

CPH mass concentration in plasma was determined at the beginning and after 60 min. The removed hemoglobin mass was calculated based on the difference between these concentrations and the plasma volume. CPH mass reduction ratios are given as percent ratio of removed CPH mass and CPH mass at the beginning of the experiment. A_405_ reduction ratios are analogously given as percent ratio between the difference of A_405_ between beginning and after 60 min and A_405_ at beginning of the experiment.

### Plasma sieving coefficients

Plasma sieving coefficients were measured according to standard ISO 8637:2010. For each test, 1 L of bovine plasma (total protein content 60 ± 5 g/L) containing hemoglobin as the solute (6 ml of freeze-thawed bovine blood added to 1 L of plasma) was recirculated at 37 °C with a QB of 300 ml/min and an UF of 60 ml/min. Samples were taken from the blood inlet, the blood outlet, and on the filtrate side. The sieving coefficients were calculated according to


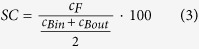


where *SC* is the sieving coefficient in [%], and *c*_*B*in,_ *c*_*B*out,_ and *c*_F_ are the solute concentrations at the blood inlet, the blood outlet, and on the filtrate side, respectively.

### Calculation of the dissociation degree

The dissociation degree is based on the assumed equilibrium between the α_2_β_2_ tetramer and 2 hemoglobin αβ dimers and is the percent ratio between the equilibrium molar dimer concentration divided by 2 and the total concentration of the tetramer. Accordingly, 100% means complete dissociation into dimers. The equilibrium molar dimer concentration was calculated using the chemical equilibrium equation that was transformed to


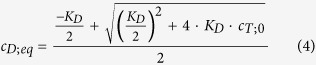


where c_D;eq_ is the dimer equilibrium concentration, K_D_ the dissociation constant, and c_T;0_ is the molar concentration of the hemoglobin tetramers.

The dissociation constants were obtained from the literature as follows: K_D_ = 5 μM, as determined by Guidotti for CO hemoglobin in 0.2 M NaCl, pH 7 and 20 °C^10^ and K_D_ = 0.2 μM, as determined by Atha and Riggs for oxyhemoglobin in 0.05 M cacodylate, 0.1 M NaCl, 1 mM EDTA, pH 7.2 and 20 °C^23^.

### Statistics

**Figure 1:**
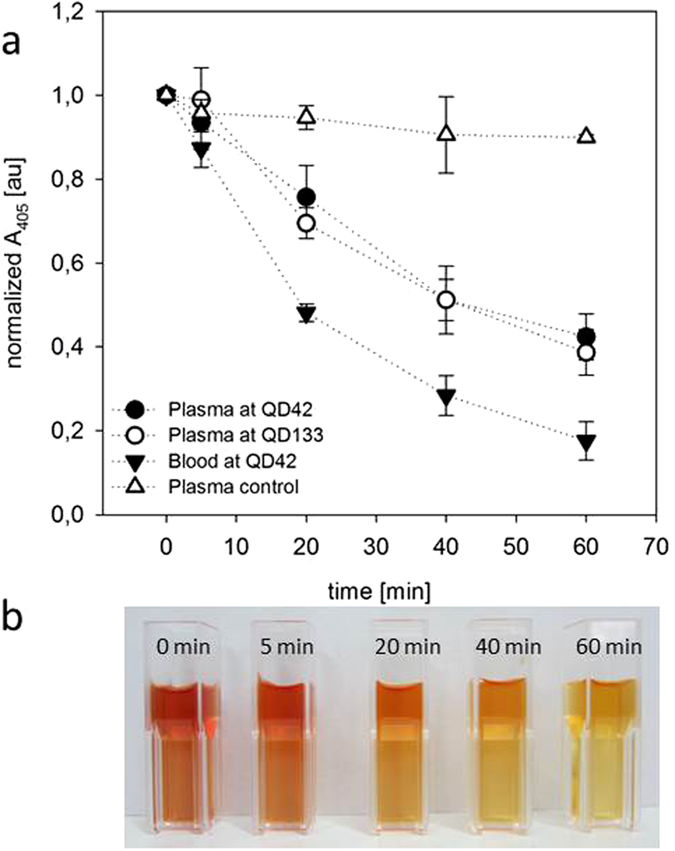
CPH removal with septeX. (**a**) Normalized A_405_ in arbitrary units (au) plotted versus time. The QB was constant at 200 ml/min and UF was 0 ml/min. QD varied and the test medium was blood or plasma. The plasma control represents plasma without added hemoglobin and treated using QB 200/QD 42/UF 0 ml/min. (**b**) Plasma samples obtained during the whole-blood treatment.

The results presented in the tables are the mean values of the results of 3 independent replicates ± the standard error of the mean. The error bars of the data points in Fig. 1 through 3, [Fig f2], [Fig f3] represent the standard error of the mean of 3 independent experiments. Replicates were always done as independent experiments with different dialyzer devices.

## Results

### Hemoglobin clearance by septeX tested with blood and plasma

Simulated dialysis treatments with septeX were conducted and CPH was determined (see Fig. 1 and Table 2). The starting CPH concentrations ranged from 668 ± 13 (plasma) to 1045 ± 75 mg/L (whole blood). CPH clearances were between 13.8 ± 1.8 and 15.5 ± 1.7 ml/min in plasma and 22.6 ± 2.9 ml/min in whole blood. The higher CPH clearances in plasma were observed at the higher QD. A steady decrease of A_405_ could be observed for each test condition, with A_405_ reduction ranging from 54 ± 5.5 to 86 ± 4.9% after 60 min. The change in color in samples obtained from the whole blood treatment illustrate the removal of CPH over time (Fig. 1b).

In simulated treatments performed in the absence of CPH (“Plasma control” in Fig. 1a), the A_405_ was decreased by approximately 10%. This decrease in absorbance cannot specifically be attributed to the decrease in CPH. Therefore, plasma control runs were taken as a baseline for the clearance calculations.

The clearance in blood was higher than the clearance in plasma under identical test conditions, possibly due to the higher viscosity of blood compared to plasma, which leads to higher internal filtration and stronger convective transfer of CPH. The concentrations of CPH were higher in whole blood because the plasma volume was reduced by the volume that was occupied by the blood cells.

The ratio of reduction determined by A_405_ and the CPH mass reduction ratio are in good agreement, supporting the assumption that the clearance calculation based on A_405_ was specifically caused by the removal of CPH.

### Hemoglobin clearance by P170H, CorDiax and MCO1-4 tested with plasma

**Figure 2 f2:**
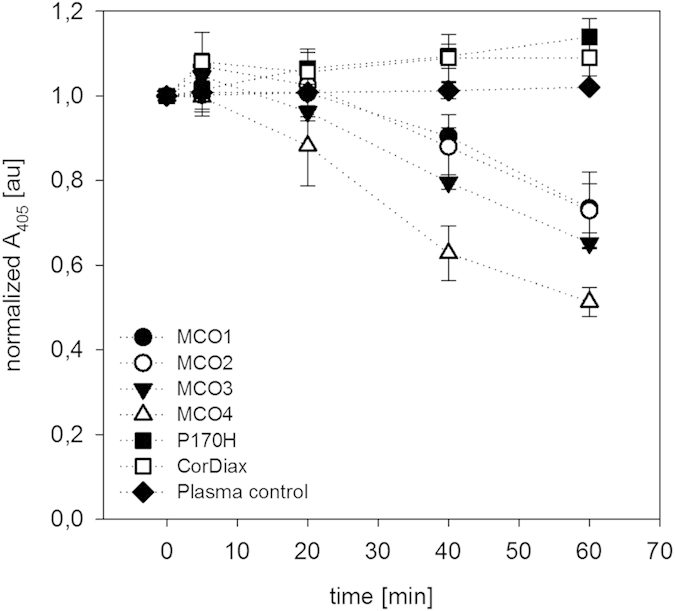
CPH removal from plasma with various filters as indicated in the key. Normalized A_405_ in arbitrary units (au) plotted versus time. The plasma control represents plasma without added hemoglobin and treated with MCO4.

Simulated dialysis treatments with P170H, CorDiax and MCO1-4 were conducted and CPH was determined (see Fig. 2 and Table 3). The starting CPH concentrations ranged from 438 ± 8.0 to 629 ± 14 mg/L. CPH clearances by MCO1-4 were between 5.8 ± 1.2 and 13.7 ± 0.8 ml/min, and CPH clearances were higher for devices with higher permeability as assessed by dextran sieving characteristics. The clearance of bovine CPH by MCO4 was 12.7 ± 1.7 ml/min which is comparable to the clearance of human CPH of 13.7 ± 0.8 ml/min. A steady decrease of A_405_ could be observed for MCO1-4, with A_405_ reduction ranging from 27 ± 5.8 to 49 ± 3.4% after 60 min.

In simulated treatments performed in the absence of CPH, the A_405_ remained constant (“Plasma control” in Fig. 2). Therefore, absorbance changes could be related directly to decreases in CPH. Plasma control runs were done with the MCO4 dialyzer that has the highest permeability of the tested high-flux dialyzers and which is representative for the other high-flux dialyzers with regard to unspecific pigment elimination.

There was a slight increase in A_405_ observed with CorDiax (9% increase) and P170H (13% increase) which can be explained by the fluid loss and no clearance was calculated in these cases.

For MCO1-4 the ratio of reduction determined by A_405_ and the CPH mass reduction ratio are in good agreement, supporting the assumption that the clearance calculation based on A_405_ was specifically caused by the removal of CPH. The mass reduction ratios were not calculated for CorDiax and P170H because no CPH removal could be detected.

### Hemoglobin clearance by P170H, CorDiax and MCO1-4 tested with blood

**Figure 3 f3:**
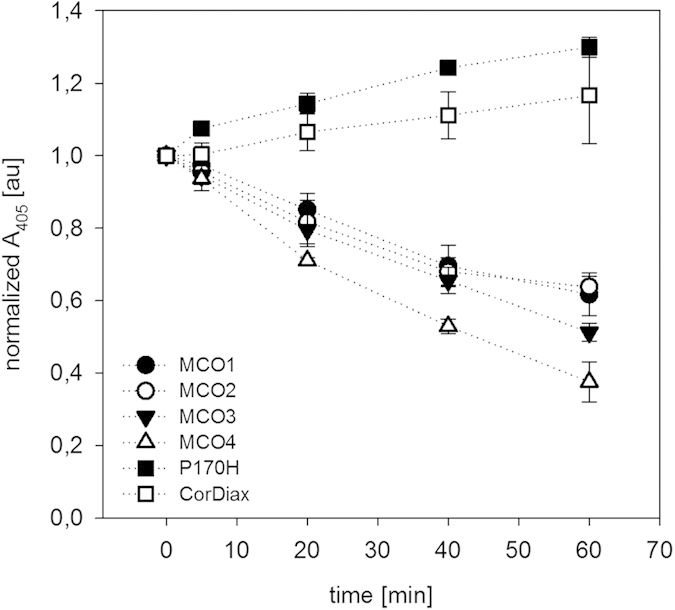
CPH removal from whole blood with various filters as indicated in the key. Normalized A_405_ in arbitrary units (au) plotted versus time.

Simulated dialysis treatments with P170H, CorDiax and MCO1-4 were conducted and CPH was determined (see Fig. 3 and Table 4). The starting CPH concentrations ranged from 820 ± 6.9 to 1186 ± 42 mg/L. CPH clearances by MCO1-4 were between 5.8 ± 1.2 and 11.3 ± 1.6 ml/min, and CPH clearances were higher for devices with higher permeability as assessed by dextran sieving characteristics. No clearance could be calculated for P170H and CorDiax.

In the case of CorDiax and P170H, there was an increase in A_405_ over time; however, for MCO1-4, A_405_ steadily decreased. Because the tests were conducted with whole blood a low degree of CPH generation might have occurred resulting from mechanical damage of erythrocytes. The site of erythrocyte damage cannot be unambiguously attributed to any part of the circuit and it is assumed that the degree of CPH generation was similar in each experiment irrespective of what dialyzer was tested. The fluid loss of about 8% from the test solution also contributes to a corresponding increase of A_405_.

The ratio of reduction determined by A_405_ and the CPH mass reduction ratio are in similar ranges, supporting the assumption that the clearance calculation based on A_405_ was specifically caused by the removal of CPH. The mass reduction ratios were not calculated for CorDiax and P170H because no CPH removal could be detected.

### Hemoglobin sieving characteristics of membranes

The CPH sieving coefficients were determined in plasma. For P170H 1.0 ± 1.0% was found. Such a low sieving coefficient indicates almost no permeability for CPH. The sieving coefficient for CorDiax could not be determined because of limited availability of those dialyzer devices. The CPH sieving coefficients of MCO1-4 were 8.8 ± 3.2% (MCO 1), 12 ± 0.8% (MCO2), 16 ± 3.5% (MCO3) and 21 ± 4.3 (MCO 4). These sieving coefficients indicate significant though limited permeability for CPH with steady increase from MCO1 to MCO4, similar to that assessed by dextran. Thus, the steady permeability increase correlates with the designed permeability of the MCO1-4 devices. The CPH sieving coefficient of septeX was found to be 35 ± 3.4%.

### Dissociation degree

**Figure 4:**
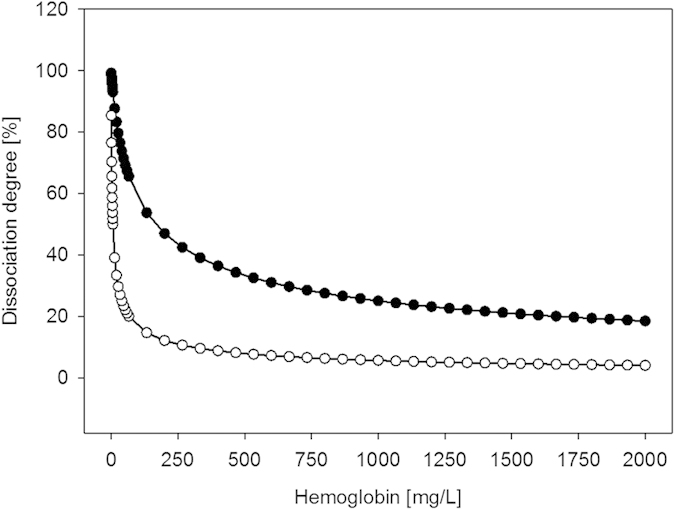
Dissociation degree of the hemoglobin tetramer into dimers plotted versus hemoglobin concentration. The curve with closed circles was calculated with K_D_ = 5 μM and the curve with open circles with K_D_ = 0.2 μM.

The degree of hemoglobin tetramer dissociation into dimers was calculated based on two dissociation constants reported in the literature: K_D_ = 5 μM^10^ and 0.2 μM^23^ for a concentration range up to 2000 mg/L (see Fig. 4). The calculation was done for defined concentrations and results are shown by circle symbols in Fig. 4; the symbols are connected by a line to indicate the actual continuous relation between hemoglobin concentration and dissociation degree. In the relevant concentration range between 250 and 1000 mg/L, the degree of dissociation is between 25 and 42% (K_D_ = 5 μM) and between 6 and 11% (K_D_ = 0.2 μM). Support for K_D_ = 5 μM can be taken from an investigation of glomerular filtration by Bunn *et al.* who reported a dissociation degree of approximately 25% for 1 g/L of hemoglobin that corresponds to K_D_ = 5 μM^24^. For the K_D_ = 0.2 μM that was reported by Atha and Riggs the presence of EDTA in the test solution might explain the lower dissociation constant because absence of bivalent ions inhibits the dissociation of hemoglobin^23^.

## Discussion

Elevated CPH concentrations are often observed as side effect of extracorporeal blood treatments but methods to remove CPH have not been clearly described. However, decreasing elevated CPH concentrations in patients’ blood remains an important target because the pathophysiological effects of elevated CPH concentrations are well described^3^,^4^,^5^,^6^,^7^.

In this study the CPH removal capacity of dialyzers with various permeability profiles was systematically investigated and it was demonstrated that dialyzers with high-flux membranes of extended permeability (MCO1-4) or with high-cutoff membranes (septeX) show significant CPH removal capacity. In contrast to that, dialyzers with conventional high-flux membranes (P170H and CorDiax) did not show significant CPH removal capacity.

CPH clearance tests with the dialyzers P170H, CorDiax and MCO1-4 and plasma as test solution were done under test conditions equal to previously published clearance data with dialyzers of extended permeability^25^,^26^. CPH clearance values in this study were below the clearance values of lambda free light chains (with molecular mass 46 kD) tested in the mentioned published study. The differences in clearance values between these studies can well be explained by the differences of the molecular weights of the solutes. Tests with whole blood were intended to simulate conditions closest to the typical clinical dialysis practice^27^.

The observed clearance values were higher than expected for CPH with a calculated molecular weight of 62.6 kD. However, 62.6 kD is the molecular weight of the tetrameric form of CPH while the dimer has 31.3 kD. The calculation of the dissociation degree suggests that between 25 and 42% of CPH is dissociated into dimers. Published work has indicated that further dissociation into monomers, which is associated with spectral changes, is hardly detectable and therefore was not considered for calculations in this study^12^,^13^. An arithmetic calculation of the apparent molecular weight of CPH considering the given dissociation into dimers resulted in an apparent molecular weight between 53.5 and 48.4 kD. Within a defined set of test parameters of this study the total CPH concentration in the experiment was sufficiently constant in order to assume that differences in clearances are the result of different membrane permeability instead of different dissociation degree of the CPH. Differences in dissociation degree might have contributed to the different clearance values between the experiments with blood and plasma; however, due to different experimental conditions it is hard to estimate how strong this contribution was. From a therapeutic perspective it might be interesting to consider that CPH is harder to remove in high concentrations because of the associated trend to form tetramers.

CPH removal in this study has been demonstrated with bovine hemoglobin and this raises the question in how far bovine hemoglobin is a suitable model for human. Guidotti reported an apparent molecular weight for human hemoglobin of 55 kD, which is less than the calculated molecular weight of 62.6 kD of the α_2_β_2_ tetramer and which is due to dissociation^10^. The apparent molecular weight of bovine hemoglobin was measured in another study to be 54 kD^28^. The reported apparent molecular weights are sufficiently similar to justify bovine hemoglobin as model compound for human hemoglobin. This justification is further supported by the finding that the CPH clearance by MCO4 is similar for bovine and human CPH (Table 3).

This study did only take hemodialysis modes without ultrafiltration into consideration. It is known that convective ultrafiltration can increase the removal capacity of high-molecular weight solutes. The low sieving coefficient of P170H, however, indicates that the conventional high-flux membranes are almost impermeably for CPH and lack of permeability seems the main reason for absence of CPH removal capacity. Comparing the clearance values against the membrane characteristics of Table 1 also shows that differences in clearance values are best reflected in pore size and size exclusion than in KoA urea and ultrafiltration coefficient which are more related to small molecule transfer and liquid permeability properties.

Acute episodes of mechanical hemolysis during ECMO treatments have been described^16^. The degree of hemolysis was classified based on CPH concentrations into the categories of absent, moderate and severe hemolysis (CPH concentrations of <0.5 g/L, 0.5–1.0 g/L and >1.0 g/L hemoglobin, respectively). 138 out of 207 patients exhibited signs of moderate to severe hemolysis. Among the hemolytic patients, 14 had severe hemolysis with CPH levels in the range of 1.18–2.05 g/L.

During acute hemolytic episodes, CPH can pass the glomerular membrane of the kidney^24^ and is then catabolized by the proximal tubule^29^. When the catabolic capacity is exceeded, hemoglobinuric acute renal failure results^7^; this failure is a potential cause for the association of adverse outcomes with hemolytic episodes during extracorporeal therapies, such as the ECMO treatments of pediatric patients. Using HD in conjunction with ECMO treatment to reduce elevated levels of CPH has been attempted; in this treatment, a Renaflo II conventional high-flux filter was used^30^. This filter was unable to reduce CPH levels; this finding highlights the insufficient hemoglobin permeability and removal capacity of conventional high-flux filters and agrees with the present findings.

For patients with end-stage renal disease, elevated CPH levels were associated with acutely blunted endothelial function, which was measured using flow-mediated dilation after a single HD session^19^. This relationship can be understood by considering the ability of hemoglobin to react with nitric oxide (NO) via heme-catalyzed oxidation^31^. This reaction reduces NO bioavailability and interferes with the paracrine signaling function of NO, adversely effecting cardiovascular function and coagulation^5^.

In conclusion, this study demonstrated the possibility of clearing CPH from blood by extracorporeal blood purification techniques using filters that have extended permeability. The combination of a septeX filter with the Prismaflex system demonstrated the highest hemoglobin removal capacity in this study and might represent a suitable choice for efficient hemoglobin removal in an acute setting and for the treatment of severe hemolytic episodes. High-flux filters with extended permeability, such as the MCO-type filters used in this study, might provide a net hemoglobin reduction in chronic dialysis settings and counteract the CPH generation that is observed as a general side effect of extracorporeal therapies. The possibility of removing CPH provides opportunities for improving patient health, as the pathophysiological effects of CPH are well described. Future clinical tests are warranted to confirm the potential applications and therapeutic efficiency of this treatment.

## Additional Information

**How to cite this article**: Hulko, M. *et al.* Cell-free plasma hemoglobin removal by dialyzers with various permeability profiles. *Sci. Rep.* **5**, 16367; doi: 10.1038/srep16367 (2015).

## Figures and Tables

**Table 1:** Membrane characteristics of dialyzers used in the study.

Dialyzer	Membrane material; Housing material	Fiber inner diameter [μm]/wall thickness [μm]	Stokes-Einstein pore radius [nm]	KoA urea [ml/min]	Ultrafiltration coefficient [ml/h x mmHg]
P170H	PAES/PVP/PA; PC	215/50	4.7	1145	70
CorDiax	PS/PVP; PP	185/md	md	1429	64
MCO1	PAES/PVP; PC	180/35	5.0	1487	48
MCO2	PAES/PVP; PC	180/35	5.4	1527	52
MCO3	PAES/PVP; PC	180/35	6.0	1662	49
MCO4	PAES/PVP; PC	180/35	6.5	1662	50
septeX	PAES/PVP; PC	215/50	10	md	md

PAES: Polyarylethersulfone; PS: Polysulfone; PA: Polyamide; PVP: Polyvinylpyrrolidone; PC: Polycarbonate; PP: Polypropylene; md: missing data.

**Table 2:** CPH removal data and CPH starting concentrations in the simulated treatment experiments using septeX filters and plasma or blood as the test medium.

Medium and flow rates [ml/min]	CPH clearance [ml/min]	CPH start concentration [mg/L]	Removed CPH mass [mg]	A_405_reduction ratio [%]	CPH mass reduction ratio [%]
Plasma QB200/QD42	13.8 ± 1.8	668 ± 13	404 ± 24	54 ± 5.5	60 ± 2.5
Plasma QB200/QD133	15.5 ± 1.7	716 ± 20	425 ± 2.6	59 ± 5.6	59 ± 1.9
Blood QB200/QD42	22.6 ± 2.9	1045 ± 75	751 ± 75	86 ± 4.9	79 ± 21

**Table 3:** CPH removal data and CPH starting concentrations of the simulated treatment experiments using various filters and plasma as the test medium.

Filter	CPH clearance [ml/min]	CPH start concentration [mg/L]	Removed CPH mass [mg]	A_405_reduction ratio [%]	CPH mass reduction ratio [%]
MCO1	5.8 ± 1.2	577 ± 21	151 ± 32	27 ± 5.8	26 ± 5.1
MCO2	7.3 ± 1.0	620 ± 13	185 ± 36	27 ± 9.0	30 ± 6.5
MCO3	8.8 ± 0.6	616 ± 7.7	234 ± 42	35 ± 0.9	38 ± 6.5
MCO4	12.7 ± 1.7 *13.7 ± 0.8	438 ± 8.0	260 ± 1.0	49 ± 3.4	59 ± 1.2
P170H	<0	629 ± 14	not determined	<0	not determined
CorDiax	<0	515 ± 85	not determined	<0	not determined

The CPH clearance of MCO4 that is marked with an asterisk was determined with human hemoglobin.

**Table 4:** CPH removal data and CPH starting concentrations of the simulated treatment experiments using various filters and whole blood as the test medium.

Filter	CPH clearance [ml/min]	CPH start concentrations [mg/L]	Removed CPH mass [mg]	A_405_reduction ratio [%]	CPH mass reduction ratio [%]
MCO1	5.8 ± 1.2	908 ± 20	268 ± 18	38 ± 10	19 ± 2.4
MCO2	5.4 ± 0.6	missing data	missing data	36 ± 5.1	missing data
MCO3	7.5 ± 0.5	820 ± 6.9	363 ± 51	49 ± 4.3	39 ± 8.6
MCO4	11.3 ± 1.6	missing data	missing data	62 ± 10	missing data
P170H	<0	1186 ± 42	not determined	<0	not determined
CorDiax	<0	970 ± 32	not determined	<0	not determined
